# Rietveld refinement of KLaTiO_4_ from X-ray powder data

**DOI:** 10.1107/S1600536811006891

**Published:** 2011-03-12

**Authors:** Bai-Chuan Zhu, Kai-Bin Tang

**Affiliations:** a Department of Chemistry, University of Science and Technology of China, Hefei, Anhui 230026, People’s Republic of China; b Department of Nanomaterial and Nanochemistry, Hefei National Laboratory for Physical Sciences at Microscale, University of Science and Technology of China, People’s Republic of China

## Abstract

Potassium lanthanum titanate(IV), KLaTiO_4_, has been synthesized by conventional solid-state reaction. It crystallizes isotypically with the Na*Ln*TiO_4_ (*Ln* = La, Pr, Nd, Sm, Eu, Gd, Y and Lu) family. Five of the six atoms in the asymmetric unit (one K, one La, one Ti and two O atoms) are situated on sites with 4*mm* symmetry, whereas one O atom has 2*mm*. site symmetry. The crystal structure can be described as being composed of single layers of distorted corner-sharing TiO_6_ octa­hedra extending parallel to (001). The layers are alternately separated by K^+^ and La^3+^ cations along [001]. The coordination number of both K^+^ and La^3+^ cations is nine, resulting in distorted KO_9_ and LaO_9_ polyhedra.

## Related literature

For the isotypic Na*Ln*TiO_4_ (*Ln* = La, Pr, Nd, Sm, Eu, Gd, Y and Lu) family, see: Toda *et al.* (1996*a* ▶). Ortho­rhom­bic symmetry for other members of this family has been reported by Nishimoto *et al.* (2006 ▶). Decomposition products of Na*Ln*TiO_4_ were investigated by Toda *et al.* (1996*b* ▶). For preparation by ion-exchange and structure analysis of K*Ln*TiO_4_ (*Ln* = La, Nd, Sm, Eu, Gd, Dy) compounds, see: Schaak & Mallouk (2001 ▶). For hydro­thermal preparation of similar compounds, see: Dairong *et al.* (1999 ▶). For crystallographic background, see: Howard (1982 ▶); Thompson *et al.* (1987 ▶).

## Experimental

### 

#### Crystal data

KLaTiO_4_
*M* *_r_* = 289.90
Tetragonal, 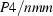

*a* = 3.84155 (10) Å
*c* = 13.4695 (4) Å
*V* = 198.78 (1) Å^3^
*Z* = 2
Cu *K*α radiation, λ = 1.54060, 1.54443 Å
*T* = 298 K
flat sheet, 20 × 20 mm

#### Data collection

PANalytical X’pert PRO diffractometer
Specimen mounting: packed powder pellet
Data collection mode: reflection
Scan method: continuous
2θ_min_ = 9.872°, 2θ_max_ = 109.815°, 2θ_step_ = 0.017°

#### Refinement

*R*_p_ = 0.046
*R*_wp_ = 0.068
*R*_exp_ = 0.046
*R*(*F* ^2^) = 0.047
χ^2^ = 2.220
5880 data points
60 parameters

### 

Data collection: *X’pert Data Collector* (PANalytical, 2003 ▶); cell refinement: *GSAS* (Larson & Von Dreele, 2004 ▶) and *EXPGUI* (Toby, 2001 ▶); data reduction: *X’pert Data Collector*; method used to solve structure: coordinates taken from an isotypic compound (Toda *et al.*, 1996*b* ▶); program(s) used to refine structure: *GSAS* and *EXPGUI*; molecular graphics: *DIAMOND* (Brandenburg, 1999 ▶); software used to prepare material for publication: *publCIF* (Westrip, 2010 ▶).

## Supplementary Material

Crystal structure: contains datablocks global, I. DOI: 10.1107/S1600536811006891/wm2446sup1.cif

Rietveld powder data: contains datablocks I. DOI: 10.1107/S1600536811006891/wm2446Isup2.rtv

Additional supplementary materials:  crystallographic information; 3D view; checkCIF report

## Figures and Tables

**Table 1:** Selected bond lengths (Å)

K1—O1	3.065 (4)
K1—O2	2.765 (9)
K1—O2^i^	2.7242 (7)
La1—O1	2.530 (3)
La1—O3^ii^	2.339 (7)
La1—O3^i^	2.7628 (12)
Ti1—O1	1.9635 (12)
Ti1—O2^iii^	1.775 (9)
Ti1—O3^iii^	2.558 (7)

Symmetry codes: (i) 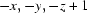; (ii) 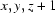; (iii) 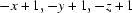.

**Fig. 1.**
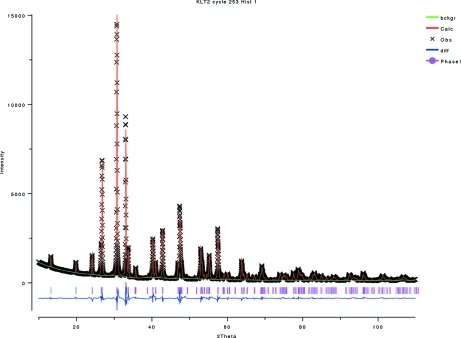
Rietveld difference plot for the refinement of KLaTiO4.

**Fig. 2.**
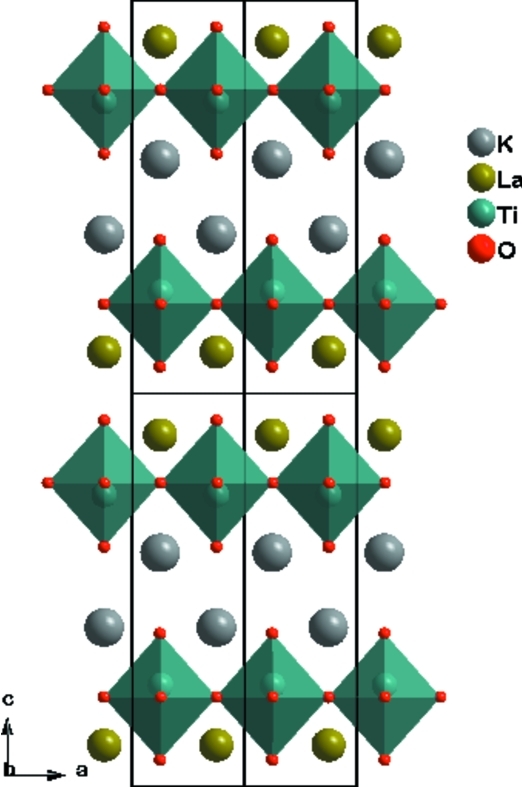
The crystal structure of KLaTiO4 in a projection along [010].

## supplementary crystallographic information

### Comment

Layered perovskites that belong to the Ruddlesden-Popper *A'*_2_[*A**_n_*_-1_*B*_2_O_3_*_n_*_+1_] familiy (*A'* = alkali, *A* = alkaline earth or rare earth cation; *B*= transition metal cation) possess a variety of interesting properties, such as superconductivity, colossal magnetoresistance, ferroelectricity, as well as catalytic activity. The structure of KLaTiO_4_ we report here is a *n* = 1 member of this familiy. Isotypic crystal structures have been reported for Na*Ln*TiO_4_ (*Ln* = La, Pr, Nd, Sm, Eu, Gd, Y and Lu; Toda *et al.*, 1996*a*) in the space group *P*4/*nmm*.

Schaak & Mallouk (2001) reported the K*Ln*TiO_4_ (*Ln*= La, Nd, Sm, Eu, Gd, Dy) family of compounds to crystallize in space group *Pbcm*, as determined from Rietveld refinements of X-ray powder data. We tested both *Pbcm* and *P*4/*nmm* space groups with the underlying structures K*Ln*TiO_4_ (*P*4/*nmm*; Schaak & Mallouk, 2001) and Na*Ln*TiO_4_ (*P*4/*nmm*; Toda *et al.*, 1996*a*) as starting models for Rietveld refinement of KLaTiO_4_. The results revealed the *P*4/*nmm* model to be significantly better than the *Pbcm* model. It is well-know that different rare earth elements can affect the crystal structure dramatically. In single layer Ruddlesden-Popper phase perovskites some studies reported that Na*Ln*TiO_4_ compounds have tetragonal symmetry for *Ln* = La—Nd, while an orthorhombic symmetry is observed for *Ln* = Sm—Lu and Y (Nishimoto *et al.*, 2006). We can infer that a similar situation might be present for K*Ln*TiO_4_ compounds. We ascribe the difference in symmetry between KLaTiO_4_ obtained through solid state reactions (tetragonal) and through ion-exchange (orthorhombic) to the different temperature treatment (higher temperatures for the solid state reaction route).

Other methods used to prepare KLaTiO_4_ have been reported previously, like an ion exchange method by Schaak & Mallouk (2001) and a hydrothermal method by Dairong *et al.* (1999). To our knowledge, a solid state route to synthesize this compound and its detailed structure analysis based on Rietveld refinement from X-ray powder diffraction data has not been reported. KLaTiO_4_ easily decomposes at high temperature and is converted into the three-layer Ruddlesden-Popper phase K_2_La_2_Ti_3_O_10_. This phenomenon is also found in during preparation of NaLaTiO_4_ reported by Toda *et al.* (1996*b*). Therefore we modified the reaction conditions on the basis of the preparation of NaLaTiO_4_ and obtained a single phase product successfully.

Fig. 1 shows the observed difference plots (calculated, observed) of the Rietveld refinement.

Fig. 2 illustrates the structure of KLaTiO_4_. It consists of a single layer of corner-sharing distorted TiO_6_ octahedra extending parallel to (001). The layers are separated by alternating layers of K^+^ and La^3+^ cations along [001]. The TiO_6_ octahedra (4*mm* symmetry) are considerably distorted. They have four equal equatorial Ti—O distances [1.9635 (12) Å], one very short Ti—O distance [1.775 (9) Å] toward the K layer and a significantly longer Ti—O distance [2.558 (7) Å] towards the La layer. The corresponding coordination polyhedra around the K^+^ and La^3+^ cations are distorted KO_9_ and LaO_9_ polyhedra, each with 4*mm* symmetry.

### Experimental

The sample was prepared by conventional solid-state reaction. The starting materials were KNO_3_, La_2_O_3_ and TiO_2_ in a molar ratio of 2:1:2. An excess of KNO_3_ (55 mol%) was added to compensate for the loss due to the volatilization of the potassium component. La_2_O_3_ was heated to 1173 K for 10 h prior to use to remove water and carbonate impurities. The mixture was then ground and calcined at 1223 K for 30 min.

### Refinement

The crystal structure of NaLaTiO_4_ (Toda *et al.*, 1996*b*) in the spacegroup *P*4/*nmm* was used as a starting model for the final Rietveld refinement of the KLaTiO_4_ structure. Isotropic displacement parameters were used for all atoms. The March-Dollase option in the *EXPGUI* program (Toby, 2001) was applied to correct for preferential orientation along [00*l*] which is often observed for such layered perovskites.

### Crystal data

**Table:**

KLaTiO_4_	*Z* = 2
*M**_r_* = 289.90	*D*_x_ = 4.848 Mg m^−^^3^
Tetragonal, *P*4/*n**m**m*	Cu *K*α radiation, λ = 1.54060, 1.54443 Å
Hall symbol: -p 4a 2a	*T* = 298 K
*a* = 3.84155 (10) Å	white
*c* = 13.4695 (4) Å	flat sheet, 20 × 20 mm
*V* = 198.78 (1) Å^3^	Specimen preparation: Prepared at 1223 K

### Data collection

**Table:**

PANalytical X'pert PRO diffractometer	Data collection mode: reflection
Radiation source: sealed tube	Scan method: continuous
graphite	2θ_min_ = 9.872°, 2θ_max_ = 109.815°, 2θ_step_ = 0.017°
Specimen mounting: packed powder pellet	

### Refinement

**Table:**

Refinement on *F*^2^	Excluded region(s): none
Least-squares matrix: full	Profile function: CW Profile function number 2 with 18 terms Profile coefficients for Simpson's rule integration of pseudovoigt function C.J. Howard (1982). J. Appl. Cryst.,15,615-620. P. Thompson, D.E. Cox & J.B. Hastings (1987). J. Appl. Cryst.,20,79-83. #1(GU) = 0.000 #2(GV) = -2.261 #3(GW) = -9.290 #4(LX) = 4.310 #5(LY) = 17.630 #6(trns) = 0.000 #7(asym) = 3.5282 #8(shft) = 0.0000 #9(GP) = 17.284 #10(stec)= 0.00 #11(ptec)= 0.00 #12(sfec)= 0.00 #13(L11) = 0.000 #14(L22) = 0.000 #15(L33) = 0.000 #16(L12) = 0.000 #17(L13) = 0.000 #18(L23) = 0.000 Peak tails are ignored where the intensity is below 0.0010 times the peak Aniso. broadening axis 0.0 0.0 1.0
*R*_p_ = 0.046	60 parameters
*R*_wp_ = 0.068	0 restraints
*R*_exp_ = 0.046	*w* = 1/[σ^2^(*F*_o_^2^) + (0.0677*P*)^2^] where *P* = (*F*_o_^2^ + 2*F*_c_^2^)/3
*R*(*F*^2^) = 0.04713	(Δ/σ)_max_ = 0.01
χ^2^ = 2.220	Background function: GSAS Background function number 1 with 36 terms. Shifted Chebyshev function of 1st kind 1: 353.285 2: -361.136 3: 220.846 4: -104.260 5: 61.8271 6: -33.1030 7: 19.7877 8: -5.01446 9: 3.42337 10: -3.14370 11: 0.340114 12: 2.15882 13: -0.130836 14: -1.88421 15: 5.08631 16: -1.48077 17: 4.42719 18: 2.91556 19: -3.924060E-0220: 0.679453 21: 5.77738 22: -2.47188 23: 3.81643 24: 3.21357 25: -4.71396 26: -1.63350 27: 0.665874 28: -7.16378 29: -7.040150E-0230: 3.04932 31: -2.36381 32: 0.787399 33: 4.27144 34: -2.96952 35: 4.90415 36: 1.54599
5880 data points	Preferred orientation correction: March-Dollase AXIS 1 Ratio= 0.96438 h= 0.000 k= 0.000 l= 1.000 Prefered orientation correction range: Min= 0.94706, Max= 1.11492

### Fractional atomic coordinates and isotropic or equivalent isotropic displacement parameters (Å^2^)

**Table:**

	*x*	*y*	*z*	*U*_iso_*/*U*_eq_	
K1	0.25	0.25	0.5950 (2)	0.0278 (9)*	
LA1	0.25	0.25	0.89446 (6)	0.0199 (4)*	
TI1	0.75	0.75	0.74203 (19)	0.0151 (7)*	
O1	0.75	0.25	0.7723 (4)	0.0189 (17)*	
O2	0.25	0.25	0.3897 (6)	0.039 (2)*	
O3	0.25	0.25	0.0681 (5)	0.018 (2)*	

### Geometric parameters (Å, °)

**Table:**

K1—O1^i^	3.065 (4)	La1—O1^iii^	2.5295 (32)
K1—O1	3.065 (4)	La1—O3^viii^	2.339 (7)
K1—O1^ii^	3.065 (4)	La1—O3^iv^	2.7628 (12)
K1—O1^iii^	3.065 (4)	La1—O3^v^	2.7628 (12)
K1—O2	2.765 (9)	La1—O3^vi^	2.7628 (12)
K1—O2^iv^	2.7242 (7)	La1—O3^vii^	2.7628 (12)
K1—O2^v^	2.7242 (7)	Ti1—O1	1.9635 (12)
K1—O2^vi^	2.7242 (7)	Ti1—O1^ix^	1.9635 (12)
K1—O2^vii^	2.7242 (7)	Ti1—O1^iii^	1.9635 (12)
La1—O1^i^	2.5295 (32)	Ti1—O1^x^	1.9635 (12)
La1—O1	2.5295 (32)	Ti1—O2^vii^	1.775 (9)
La1—O1^ii^	2.5295 (32)	Ti1—O3^vii^	2.558 (7)
			
O1^i^—K1—O1	77.62 (13)	O1^i^—La1—O3^v^	65.85 (12)
O1^i^—K1—O1^ii^	52.61 (8)	O1^i^—La1—O3^vi^	130.30 (11)
O1^i^—K1—O1^iii^	52.61 (8)	O1^i^—La1—O3^vii^	130.30 (11)
O1^i^—K1—O2	141.19 (6)	O1—La1—O1^ii^	64.95 (9)
O1^i^—K1—O2^iv^	59.94 (15)	O1—La1—O1^iii^	64.95 (9)
O1^i^—K1—O2^v^	59.94 (15)	O1—La1—O3^viii^	130.59 (9)
O1^i^—K1—O2^vi^	112.52 (18)	O1—La1—O3^iv^	130.30 (11)
O1^i^—K1—O2^vii^	112.52 (18)	O1—La1—O3^v^	130.30 (11)
O1—K1—O1^ii^	52.61 (8)	O1—La1—O3^vi^	65.85 (12)
O1—K1—O1^iii^	52.61 (8)	O1—La1—O3^vii^	65.85 (12)
O1—K1—O2	141.19 (6)	O1^ii^—La1—O1^iii^	98.81 (17)
O1—K1—O2^iv^	112.52 (18)	O1^ii^—La1—O3^viii^	130.59 (9)
O1—K1—O2^v^	112.52 (18)	O1^ii^—La1—O3^iv^	65.85 (12)
O1—K1—O2^vi^	59.94 (15)	O1^ii^—La1—O3^v^	130.30 (11)
O1—K1—O2^vii^	59.94 (15)	O1^ii^—La1—O3^vi^	65.85 (12)
O1^ii^—K1—O1^iii^	77.62 (13)	O1^ii^—La1—O3^vii^	130.30 (11)
O1^ii^—K1—O2	141.19 (6)	O1^iii^—La1—O3^viii^	130.59 (9)
O1^ii^—K1—O2^iv^	59.94 (15)	O1^iii^—La1—O3^iv^	130.30 (11)
O1^ii^—K1—O2^v^	112.52 (18)	O1^iii^—La1—O3^v^	65.85 (12)
O1^ii^—K1—O2^vi^	59.94 (15)	O1^iii^—La1—O3^vi^	130.30 (11)
O1^ii^—K1—O2^vii^	112.52 (18)	O1^iii^—La1—O3^vii^	65.85 (12)
O1^iii^—K1—O2	141.19 (6)	O3^viii^—La1—O3^iv^	79.48 (14)
O1^iii^—K1—O2^iv^	112.52 (18)	O3^viii^—La1—O3^v^	79.48 (14)
O1^iii^—K1—O2^v^	59.94 (15)	O3^viii^—La1—O3^vi^	79.48 (14)
O1^iii^—K1—O2^vi^	112.52 (18)	O3^viii^—La1—O3^vii^	79.48 (14)
O1^iii^—K1—O2^vii^	59.94 (15)	O3^iv^—La1—O3^v^	88.09 (5)
O2—K1—O2^iv^	94.34 (18)	O3^iv^—La1—O3^vi^	88.09 (5)
O2—K1—O2^v^	94.34 (18)	O3^iv^—La1—O3^vii^	158.96 (27)
O2—K1—O2^vi^	94.34 (18)	O3^v^—La1—O3^vi^	158.96 (27)
O2—K1—O2^vii^	94.34 (18)	O3^v^—La1—O3^vii^	88.09 (5)
O2^iv^—K1—O2^v^	89.672 (27)	O3^vi^—La1—O3^vii^	88.09 (5)
O2^iv^—K1—O2^vi^	89.672 (27)	O1—Ti1—O1^ix^	156.05 (33)
O2^iv^—K1—O2^vii^	171.3 (4)	O1—Ti1—O1^iii^	87.53 (7)
O2^v^—K1—O2^vi^	171.3 (4)	O1—Ti1—O1^x^	87.53 (7)
O2^v^—K1—O2^vii^	89.672 (27)	O1—Ti1—O2^vii^	101.97 (16)
O2^vi^—K1—O2^vii^	89.672 (27)	O1^ix^—Ti1—O1^iii^	87.53 (7)
O1^i^—La1—O1	98.81 (17)	O1^ix^—Ti1—O1^x^	87.53 (7)
O1^i^—La1—O1^ii^	64.95 (9)	O1^ix^—Ti1—O2^vii^	101.97 (16)
O1^i^—La1—O1^iii^	64.95 (9)	O1^iii^—Ti1—O1^x^	156.05 (33)
O1^i^—La1—O3^viii^	130.59 (9)	O1^iii^—Ti1—O2^vii^	101.97 (16)
O1^i^—La1—O3^iv^	65.85 (12)	O1^x^—Ti1—O2^vii^	101.97 (16)

Symmetry codes: (i) *x*−1, *y*, *z*;  (ii) −*y*+1/2, *x*−1, *z*; (iii) −*y*+1/2, *x*, *z*; (iv) −*x*, −*y*, −*z*+1; (v) −*x*, −*y*+1, −*z*+1; (vi) −*x*+1, −*y*, −*z*+1; (vii) −*x*+1, −*y*+1, −*z*+1; (viii) *x*, *y*, *z*+1; (ix) *x*, *y*+1, *z*; (x) −*y*+3/2, *x*, *z*.
